# Knockdown of lncRNA-ASLNC12002 alleviates epithelial–mesenchymal transition of type II alveolar epithelial cells in sepsis-induced acute respiratory distress syndrome

**DOI:** 10.1007/s13577-022-00837-8

**Published:** 2022-12-07

**Authors:** Kaixuan Feng, Weifeng Huang, Jiawei Shang, Feng Ping, Qin Tan, Wei Wang, Yingchuan Li, Yongmei Cao

**Affiliations:** 1 Department of Critical Care Medicine, Jinshan Branch of Shanghai Sixth People’s Hospital, Shanghai, China; 2 Department of Critical Care Medicine, Shanghai Jiaotong University Affiliated The Sixth People’s Hospital, Shanghai, China; 3 Department of Critical Care Medicine, School of Medicine, Shanghai Tenth People’s Hospital, Tongji University, Shanghai, China

**Keywords:** Sepsis, Acute respiratory distress syndrome, Snail1, Epithelial–-mesenchymal transition, Alveolar epithelial cells, ASLNC12002

## Abstract

Patients with sepsis-induced acute respiratory distress syndrome (ARDS) have higher mortality and poor prognosis than pneumonia-induced ARDS. Pulmonary fibrosis is an irreversible accumulation of connective tissue in the interstitium of the lung and closely associated with the epithelial–mesenchymal transition (EMT) of type II alveolar epithelial cells (AECIIs). Therefore, it is undoubtedly worth studying whether the EMT of AECIIs in sepsis-induced ARDS patients is different from that in patients with pneumonia-induced ARDS in the regulatory mechanism. Here, we will report for the first time that an lncRNA-ASLNC12002 is highly expressed in AECIIs of patients with sepsis-induced pneumonia and promotes EMT in AECIIs. The research results showed that the expression of ASLNC12002 in AECIIs derived from patients with sepsis-induced ARDS is significantly higher than that in normal people and pneumonia-induced ARDS patients. Mechanism research showed that ASLNC12002 can cause the inactivation of the anti-EMT pathway NR2F2/miR128-3p/Snail1 by acting as the sponge of miR128-3p. Functional experiments showed that targeted silencing of ASLNC12002 could effectively inhibit EMT progression in AECIIs of patients with sepsis-induced pneumonia by restoring NR2F2/miR128-3p/Snail1 pathway. In a word, our study shows for the first time that the inactivation of NR2F2/miR128-3p/Snail1 pathway caused by the enhanced expression of ASLNC12002 is the direct reason why AECIIs in sepsis-induced ARDS patients are prone to get EMT progress. ASLNC12002 has the potential to become a biological target for the prevention and treatment of pulmonary fibrosis in patients with sepsis-induced ARDS. At the same time, the expectation that ASLNC12002 and its related products may be used as clinical markers for the evaluation of early pulmonary fibrosis in ARDS patients should not be ignored.

**Supplementary Information:**

The online version contains supplementary material available at 10.1007/s13577-022-00837-8.

## Introduction

Sepsis, a life-threatening organ dysfunction caused by various factors, is widely concerned by medical researchers all over the world. More than 18 million people suffer from sepsis and there are almost 250,000 deaths per year [1, 2]. Lung is the most vulnerable target organ after sepsis and sepsis-induced lung injury manifests as acute respiratory distress syndrome (ARDS) [3, 4]. It is confirmed that the early stage of ARDS is characterized by inflammatory response, accompanied by rapid fibroproliferative response [5]. Studies have shown that pulmonary fibrosis is the leading cause of poor prognoses in patients with sepsis-induced ARDS, and epithelial-to-mesenchymal transition (EMT) is the pivotal pathological process of pulmonary fibrosis [6, 7]. In our previous study, we found that the inflammation response and pulmonary fibrosis significantly increased in an animal model of lipopolysaccharide (LPS)-induced early pulmonary fibrosis and that the scores of lung injury and pulmonary fibrosis in the LPS group were significantly higher than those of the control group, suggesting that LPS causes significant lung injury and early pulmonary fibrosis [8]. Meanwhile, our research also showed that following exposure to LPS, epithelial cells and alveolar structure were damaged. What's more, type II of alveolar epithelial cells (AECIIs) undergoing EMT was evidenced by decreased expression of epithelial markers (E-Cadherin), and increased expression of mesenchymal markers (α-SMA and Vimentin) [9]. Therefore, exploring the mechanism of EMT in AECIIs of patients with sepsis-induced ARDS will be of great significance for solving clinical problems and improving prognosis.

Snail1 is the founding member of the Snail superfamily of zinc-finger transcription factors and plays a key role in transcriptional regulation, formation of chromatin structure, regulation of cell cycle and cell survival, promotion of embryonic development and migration, and growth and invasion of tumor [10]. Studies also have revealed that snail1 was essential for triggering EMT and suppresses tumor cell migration in a human lung cancer cell line [11]. Intriguing, we noticed that long non-coding RNAs (lncRNAs) have been proved to be closely related to a variety of biological functions [12, 13]. Growing evidence indicates that lncRNAs are key modulators of various physiological and pathological processes in disease states, including cell proliferation, invasion, migration, and apoptosis [14, 15]. In recent years, studies have shown that lncRNA CASC9 [16], TUG1 [17], H19 [18], and MALAT1 [19] are associated with sepsis-induced lung injury and pulmonary inflammatory response. However, it is unknown about ASLNC12002 in regulating pulmonary fibrosis during sepsis-derived ARDS. ASLNC12002 obtained through high-throughput screening is located on human chromosome 2 (GRCh38.p13), which is the key lncRNA highly expressed in AECIIs of patients with sepsis-induced ARDS. We wondered if ASLNC12002 involves in EMT and sepsis-induced early pulmonary fibrosis as well as the potential role it may play.

Thus, in this study, we investigated the impact and mechanisms by which ASLNC12002 is involved in the process of EMT in AECIIs of patients with sepsis-induced ARDS. These findings indicate that ASLNC12002 may provide a theoretical and experimental basis for the development of effective treatment strategies for patients with sepsis-induced ARDS.

## Materials and methods

### Sorting and identification of primary AECIIs

48 patients (16 patients without pulmonary disease, 16 patients with pneumonia-induced ARDS, and 16 patients with sepsis-induced ARDS), male or female, aged 18–85 y, were recruited in the Department of Critical Care Medicine in Shanghai Jiao Tong University Affiliated Sixth People’s Hospital. The study was approved in accordance with the standards by the Ethics Committee of at the College of Medicine, Shanghai Jiao Tong University (approval number 2020-KY-087) and informed consent was obtained from all patients (Table 1).

**Table 1:** Clinical features of 48 patients

Group	Gender	Age (y)	APACHE II score	PaO_2_/FiO_2_ (mmHg)
Normal	Male	82	11	310
	Male	39	2	429
	Female	64	5	386
	Male	89	13	319
	Female	72	9	329
	Female	67	8	338
	Female	54	4	324
	Male	75	7	310
	Male	51	6	471
	Female	41	3	485
	Male	65	8	424
	Male	57	7	433
	Female	69	9	343
	Female	72	12	314
	Female	58	6	371
	Male	77	8	333
16 patients with pneumonia-induced ARDS	Male	66	12	198
	Female	34	7	256
	Male	85	15	142
	Male	72	9	147
	Male	69	8	200
	Female	65	8	178
	Female	69	10	188
	Female	84	14	196
	Female	64	7	206
	Male	67	8	194
	Male	83	12	167
	Male	75	10	158
	Male	40	5	208
	Female	66	9	147
	Female	87	14	150
	Female	68	8	152
16 patients with sepsis-induced ARDS	Male	85	13	190
	Female	84	10	153
	Male	95	16	148
	Male	84	12	196
	Male	83	10	165
	Male	45	7	200
	Female	47	8	206
	Female	70	10	216
	Female	76	12	194
	Male	65	9	202
	Male	77	13	145
	Female	58	9	204
	Female	54	7	172
	Female	87	11	212
	Male	59	9	175
	Female	90	17	147

The patients were routinely prepared, 2% lidocaine (15 mL) was used for anesthesia, and bronchoalveolar lavage was performed with 0.9% sterile normal saline (100 mL of saline solution sequentially instilled and suctioned in 20 mL portions) at 37 °C using electronic bronchoscopy. BALF samples were obtained from a subsegment of the right middle lobe of the lung and recovered at a rate of 30–50% according to the procedure described in the 2012 American Thoracic Society guideline. BALF was packed in Eppendorf tube and centrifuged at 1000 × *g* for 10 min at 4 °C to remove mucus and cells. The supernatants were aliquoted and frozen at − 80 °C until analysis.

Bronchoalveolar lavage fluids (BALF), were collected from all patients and used for obtaining primary AECIIsby flow cytometry. Cells of double-labeled (pan cytokeratin + /EpCAM +) were defined as AECIIs.

### Cell culture

Primary AECIIs were cultured in RPMI1640 medium (Thermofisher, CA, USA) supplemented with 10% fetal bovine serum (FBS, Thermofisher). 293 T cells, purchased from American Type Culture Collection (ATCC, VA, USA) and cultured in DMEM medium (Thermofisher) supplemented with 10%FBS, were used for the generation of lentiviruses and luciferase experiments. All adherent cells were cultured in a humidified atmosphere of 5% CO_2_ at 37 °C and passaged by digestion with 0.25% trypsin (Thermofisher) once the cells reached a density of 75%.

### Vector construction and recombinant lentivirus preparation

The coding sequence (CDS, 1246 bp) of the human Nuclear Receptor Subfamily 2 Group F Member 2 (NR2F2) gene (NM_021005.4) was amplified by PCR using human complementary DNA(cDNA) as the template with the primers 5’-GGAATTCGCCACCATGGCAATGGTAGTCAGCACGTGG-3’ (forward) and 5’-CGGGATCCTTATTGAATTGCCATATACGGCCAG-3’ (reverse). The product was digested and cloned into the expression vector to construct pcDH-NR2F2. PcDH-ASLNC12002 was also constructed using the same method with the PCR primers 5’-GGAATTCTTCCTCTAGAAATGGAATAGCAATG-3’ (forward) and 5’-CGGGATCCAATAGATCTCTCATTTGCTACTTAC-3’ (reverse). An siRNA (5’-GACTTCCAGAGTCAAGCTT-3’) that bound complementarily to ASLNC12002 was chosen and used for construction of pSIH1-shRNA-ASLNC12002. A nonsense sequence of siRNA (5’-AACTTTGATCGCGCAGCTA-3’) was used as negative control (NC) for construction of pSIH1-NC. All the recombinant vectors were sequenced, and endotoxin-free plasmids were prepared using an EndoFree Plasmid Kit (Qiagen, Germany).

A total of 1 × 10^6^ 293 T cells in logarithmic growth phase were plated into 10-cm dishes in 10-mL DMEM containing 10% FBS and cultured overnight. Two micrograms of pSIH1-shRNA-ASLNC12002 or pSIH1-NC) and 10 μg of the pPACK Packaging Plasmid Mix (System Biosciences, CA, USA) were co-transfected using t Lipofectamine 2000 (thermofisher). The medium was fully replaced with DMEM plus 2% FBS before transfection. After 48 h of transfection, the supernatants were harvested and cleared by centrifugation at 5000 × *g* at 4 °C for 10 min and then passed through a 0.45 µm PVDF membrane (Millipore, MI, USA). The titer of the virus was determined by a gradient dilution method. The recombinant lentiviruses were named Lv-shRNA-ASLNC12002 and Lv-NC.

### Verification of the binding sites of hsa-miR128-3p on 3’-UTR of Snail1 mRNA

Primers that targeted the 3’-UTR of Snail1 gene were designed, such that flanking XbaI restriction sites were introduced into the PCR product containing the miR128-3p target site (5’-ACUGUGA-3’).

Bioinformatics prediction software TargetScan7.1 (Whitehead Institute for Biomedical Research, MI, USA) was used to predict the potential binding sites of miR128-3p in the 3’-UTR of Snail1 mRNA. The PCR primers forward 5’-GCTCTAGACCACGAGGTGTGACTAACTATG-3’ and reverse 5’-GCTCTAGACAAGTGACAGCCATTACTCACAG-3’ targeting the 3’-UTR of Snail1 were designed and used to introduce the PCR product containing the miR1283p-binding sites (5’-ACUGUGA-3’) which was used to construct the luciferase vector pGL3-wt (wild-type)-Snail1. Then, the miR128-3p target sites in the pGL3-wt-Snail1 vector were mutated from 5’-ACUGUGA-3’ to 5’-UGGUACA-3’ to construct the mutated vector pGL3-mt-Snail1 by a Site-Directed Mutagenesis Kit (Takara Bio.). The miR128-3p mimic (5’-UCACAGUGAACCGGUCUCUUUtt-3’), inhibitor (5’- AAAGAGACCGGUUCACUGUGAtt-3’), and NC (5’-CUGAAUUCUUACUACCCGGGUtt-3’) were all chemically synthesized (Sangon, Shanghai, China). 293 T cells were co-transfected with the miR128-3p mimic, inhibitor, NC, and pGL-wt-Snail1 or pGL3-mt-Snail1 using Lipofectamine 2000. Then, the cells were harvested, and luciferase assays were performed using a Dual Luciferase Reporter Assay System (Promega Corporation, WI, USA) 48 h after transfection. The effect of ASLNC12002 depletion on the inhibition of luciferase activity by the miR128-3p mimic was evaluated in 293 T cells using Dual Luciferase Reporter Assay System. In addition, the effect of exogenous ASLNC12002 on inhibition of luciferase by the miR128-3p mimics was evaluated in 293 T cells co-transfected with miR128-3p mimics and pGL3-wt-Snail1 and pcDH-ASLNC12002.

### Validation of the transcription factor-binding site (TFBS) of NR2F2 in the promoter region miR128-3p

The location of miR128-3p precursor in the human genome was searched for and a 2.5 kb DNA sequence upstream of the transcription start site was selected as the promoter region. Then, the promoter sequence was predicted using Promoter 2.0 software (http://www.cbs.dtu.dk/services/Promoter). A TFBS for NR2F2 in the miR128-3p promoter (312 bp) was predicted by “JASPAR” (http://genexplain.com/transfac). The predicted promoter of miR128-3p was amplified using human genomic DNA as the template and cloned into the luciferase reporter vector pGL3-Enhancer (Promega Corporation) at the upstream of the luciferase gene to construct pGL3-wt (TFBS)-miR128, which carried the wild-type TFBS. The PCR primers were: 5’-GGGGTACCCTTGAACTTAAAGAAATTTTGCC-3’(forward) and 5’-CCCAAGCTTGGTATGGAGTTTTACAGTACACAGAG-3’(reverse). Then, the TFBS in the pGL3-wt (TFBS)-miR128 was mutated from 5’-GGGAAATATCT-3’ to 5’-TATCCAGAGATG-3’ to construct pGL3-mt (TFBS)-miR128. The experimental groups: control group (without transfection), pGL3-wt (TFBS)-miR128 transfection group, pGL3-mt (TFBS)-miR128 transfection group, pGL3-wt(TFBS)-miR128 + pcDH-ASLNC12002 co-transfection group, and pGL3-mt (TFBS)-miR128 + pcDH-ASLNC12002 co-transfection group. Luciferase experiments were carried out in 293 T cells using Lipofectamine 2000 according to the manufacturer’s instructions. After 48 h of transfection, the cells were harvested and used for luciferase assays. Then, the miR128-3p promoter sequence was cloned to the upstream of the green fluorescent protein (GFP) gene to construct a fluorescent expression vector which was transfected into 293 T cells, and the promoter activity and efficiency were evaluated by GFP expression in 293 T cells 48 h after transfection.

### Chromatin immunoprecipitation-PCR (Chip-PCR)

AECIIs from patients with sepsis-induced ARDS were harvested and subjected to Chip-PCR using EZ CHIP Kit (Millipore, MI, USA) in accordance with the manufacturer’s protocol. The primers for RT-PCR were: 5’-CCTTTGGTGGATAGGGAGGAGG-3’(forward) and 5’- GGATAAAAGACCTAGCATTTTATCC-3’ (reverse). The experiment obtained a product of 58 bases carrying the predicted TFBS of NR2F2, which was cloned into a simple T-vector (Takara) for sequencing. NR2F2 primary antibody (2 μg; Abcam, Cambridge, UK) was used for immunoprecipitation of the target protein.

### RNA-binding protein immunoprecipitation (RIP) assay

We identified the binding of Snail1 to miR128-3p or ASLNC12002 in AECIIs by RIP experiments. Snail1 protein in AECIIs was precipitated by Immunoprecipitation (IP), and its bound RNA was obtained from the eluent. Then, the target sequence belongs to ASLNC12002 or mature miR128-3p in the purified product was qualitatively detected by reverse transcription-PCR (RT-PCR) with the TaKaRa PrimeScriptTM One Step RT-PCR Kit (Takara, Dalian, China). Two micrograms of Snail1 primary antibody (Abcam, Cambridge, UK) were used for IP, and the reverse transcription primers and PCR primers were the same as those of real-time fluorescence quantitative PCR (RT-qPCR). The theoretical PCR product carrying the predicted sequence was analyzed by 2% agarose gel electrophoresis.

### Cell proliferation, invasion, and apoptosis assay

AECIIs were infected with the recombinant lentiviruses (Lv-NC and Lv-shRNA-ASLNC12002) at a multiplicity of infection (MOI) of 10. Cell viability was detected using a cell counting kit-8 assay (CCK-8, Dojindo, Japan) at 24, 48, and 72 h in accordance with the manufacturer’s protocol. Cell invasion experiments were performed using the QCMTM 24-well Fluorimetric Cell Invasion Assay kit (ECM554, Chemicon International, WI, USA) according to the manufacturer’s instructions. Cells with the same treatment were collected and used for apoptosis assay using flow cytometry (FACS Calibur, BD Biosciences, NJ, SUA) with treatment of Annexin V: FITC Apoptosis Detection Kit II (BD Biosciences). Cells were digested by trypsinization and washed with dPBS and suspended in 500 μl binding buffer and added with 5 μL Annexin V-FITC in dark for 10 min and then were stained with 5 μl Propidium Iodide for 5 min. Apoptosis was analyzed on BD-FACS Calibur using FITC (FL1) channel and PI (FL2) channel at an excitation wavelength at 488 nm.

### Quantitative reverse transcription-PCR (RT-qPCR)

Total RNA was isolated and reverse-transcribed into cDNA using M-MLV reverse transcriptase. Real-time PCR was performed using the SYBR Premix Ex Taq™ kit and TP800 System (Takara) under the following the manufacturer's instructions. The PCRs were carried out under the following conditions: 40 cycles of denaturation at 95 °C for 10 s, annealing at 60 °C for 20 s, and extension at 72 °C for 20 s, and cDNA (200 ng) was used as the template. For the determination of the miR128-3p and ASLNC12002 levels, U6 snRNA was used as the reference. The specific primers used for reverse transcription were 5’-TACCTTGCGAAGTGCTTAAAC-3’ for U6 snRNA and 5’-GTCGTATCCAGTGCGTGTCGTGGAGTCGGCAATTGCACTGGATACGAAGTGT-3’ for miR128-3p.

The PCR primers were: Snail1, 5’-GCCGTGCCTTCGCTGAC-3’ (forward) and 5’-ACGCCTGGCACTGGTACTTCTTGA-3’ (reverse); α-SMA,5’-CCACCACCACCCCACTTACTATCA-3’ (forward) and 5’-GTGGTGGGGGAATTATTGGTGGTC-3’ (reverse); Vimentin,5’-TGACCGCTTCGCCAACTACAT-3’ (forward) and 5’-CTCGGCCAGCAGGATCTTATTCT-3’ (reverse); E-cadherin,5’-AGCCCCGCCTTATGATTCTCTG-3’ (forward) and 5’-AACCGCTTCCTTCATAGTCAAACA-3’ (reverse);β-actin, 5’-CCTGTACGCCAACACAGTGC-3’ (forward) and 5’-ATACTCCTGCTTGCTGATCC-3’ (reverse); U6 snRNA, 5’-GTGCTCGCTTCGGCAGCACAT-3’ (forward) and 5’-TACCTTGCGAAGTGCTTAAAC-3’ (reverse); miR128-3p, 5’-TTTCUCTGGCCAAGTGACACT-3’ (forward) and 5’-AAGCTGCCAGTTGAAGAACTGT-3’(reverse). ASLNC12002, 5’-TTCCCACGACCCTCAGCCTGCC-3’ (forward) and 5’-TTCCGCCAGGACCTGCGGAGGC-3’ (reverse). The levels of Snail1, α-SMA, Vimentin, and E-cadherin were normalized to the expression of an endogenous housekeeping gene, β-actin, using the 2^−ΔCt^ method. For the detection of the miR128-3p and ASLNC12002 levels, U6 snRNA was used as the reference. In addition, we also separated the cytoplasm of AECIIs through a Cytoplasmic & Nuclear RNA Purification Kit (Norgen Biotek, Thorold, ON, Canada), which were used for detecting the proportion of ASLNC12002 in the cytoplasm by RT-qPCR with the total RNA of AECIIs as the reference.

### Western blotting

The total protein was extracted from cells using the M-PER mammalian protein extraction reagent (Pierce, Rockford, IL, USA) in accordance with the manufacturer’s instructions. Equal amounts of total protein (10 μg) were loaded onto SDS-PAGE gels (11%) and transferred onto nitrocellulose membranes. The blots were probed at 4 °C overnight with the primary antibodies against human NR2F2(1:400), Snail1 (1:500), α-SMA (1:400), Vimentin (1:450), E-cadherin (1:400), and β-actin (1:1000) (Abcam), followed by probing with the secondary HRP-conjugated anti-rabbit/mouse antibody (1:4000 dilution) at room temperature for 2 h. After washing, the bands were detected by chemiluminescence and imaged with X-ray films. β-Actin was used as an endogenous reference for normalization.

### Statistical analysis

The data are shown as the mean ± s.d. of three independent experiments. All statistical data were analyzed using SPSS Statistics GradPack version 20.0 software (IBM Corp., Armonk, NY, USA) and GraphPad Prism 7.0 (GraphPad Software, Inc., La Jolla, CA, USA). Comparisons between groups were analyzed using a two-tailed Student’s *t*-test or one-way ANOVA with a post hoc Tukey test. A value of *P* < 0.05 was considered statistically significant.

## Results

### AECIIs show obvious EMT in patients with sepsis-induced ARDS

**Fig. 1:**
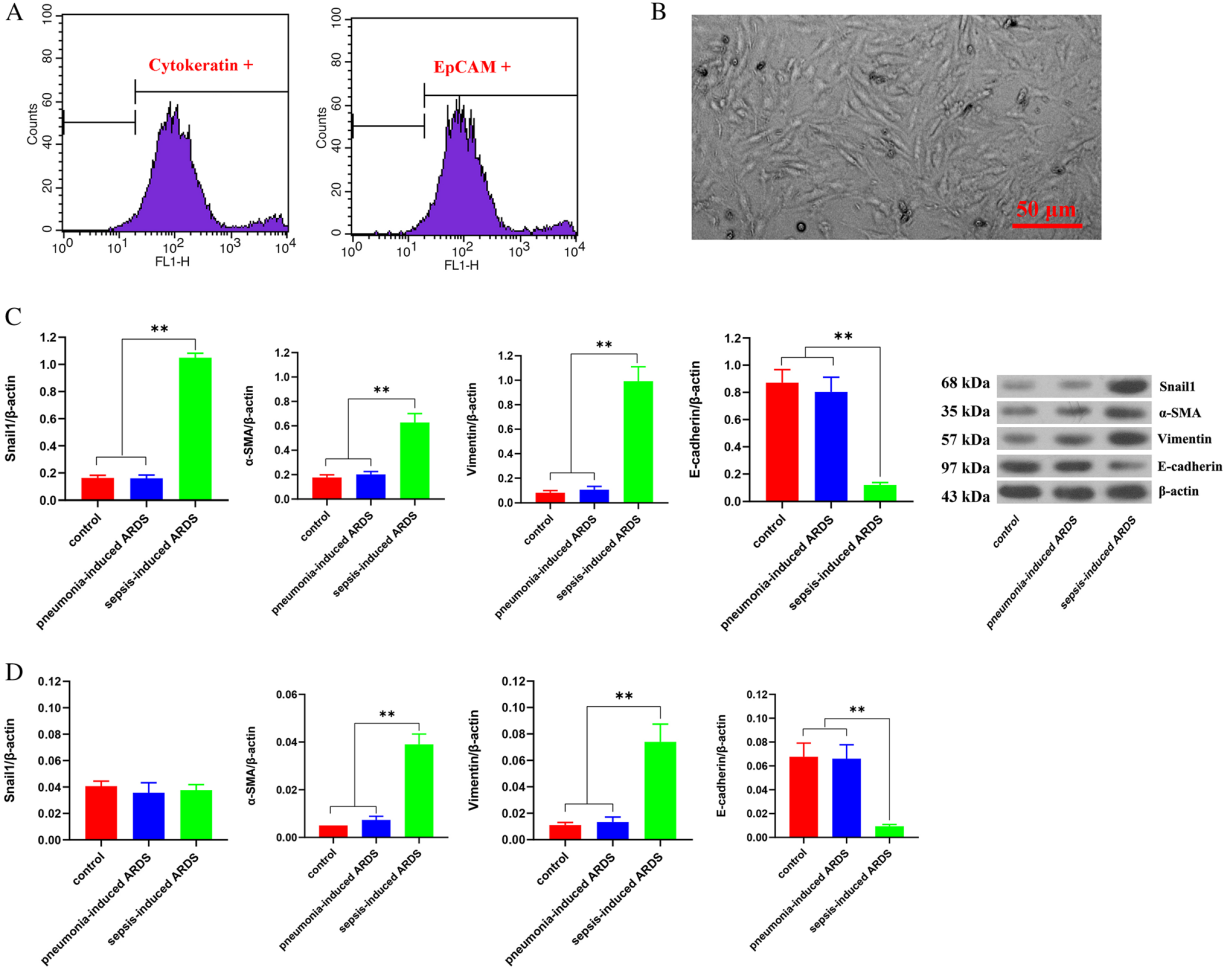
Preparation of primary AECIIs and evaluation of their EMT characteristics **A** Identification of primary AECIIs (Cytokeratin + /EpCAM) by flow cytometry. Horizontal axis indicates the signal intensity of the marker proteins, and vertical axis indicates the number of AECIIs. **B** Morphological observation of primary AECIIs by an inverted microscope. **C** The expression of proteins Snail1, Vimentin, α-SMA, and E-cadherin in AECIIs were detected by western blotting. β-Actin served as an internal reference. **D** The mRNA levels of Snail1, Vimentin, α-SMA, and E-cadherin in AECIIs were detected by RT-qPCR. β-Actin served as an internal reference, and the 2^−ΔCt^ method was used to analyze inter-group differences. All data are expressed as the mean ± s.d. from at least three replicate experiments. **P < 0.01

The results of flow cytometry showed that the purity of AECIIs was not less than 96% (Fig. 1A). Morphological observations showed that AECIIs of patients with sepsis-induced ARDS had significant characteristics of interstitial cells (Fig. 1B). Western blotting results demonstrated that the Snail1, α-SMA, and Vimentin protein expressions in AECIIs of patients with sepsis-induced ARDS were upregulated markedly, while the expression of E-cadherin was lower than those of control group and pneumonia-induced ARDS group (*P* < 0.01). There were no significant differences in protein expression of Snail1, α-SMA, Vimentin, and E-cadherin between control group and pneumonia-induced ARDS group (*P* > 0.05) (Fig. 1C). The RT-qPCR data showed that the mRNA levels of α-SMA and Vimentin in sepsis-induced ARDS group were lower, but E-cadherin was higher than those of control group and pneumonia-induced ARDS group (*P* < 0.01). There were no significant differences in mRNA levels of α-SMA, Vimentin, and E-cadherin between control group and pneumonia-induced ARDS group (*P* > 0.05). The important finding is that Snail1 mRNA levels were not significantly different among the three groups (*P* > 0.05) (Fig. 1D). These results indicated that AECIIs had obvious EMT characteristics in patients with sepsis-induced ARDS. The expression changes of EMT related functional gene α-SMA, Vimentin and E-cadherin were highly consistent at the transcriptional and post-transcriptional levels in different groups. However, the abnormal expression of Snail1 only occurs at the protein level, not at the mRNA level in AECIIs of patients with sepsis-induced ARDS. However, the increase of the Snail1 is limited at the level of protein, not at the mRNA level in AECIIs of patients with sepsis-induced ARDS.

### MiR128-3p can negatively regulate the expression of Snail1 protein by binding to the 3'UTR region of its mRNA, which is only maintained in AECIIs from normal people and patients with sepsis-induced ARDS

**Fig. 2:**
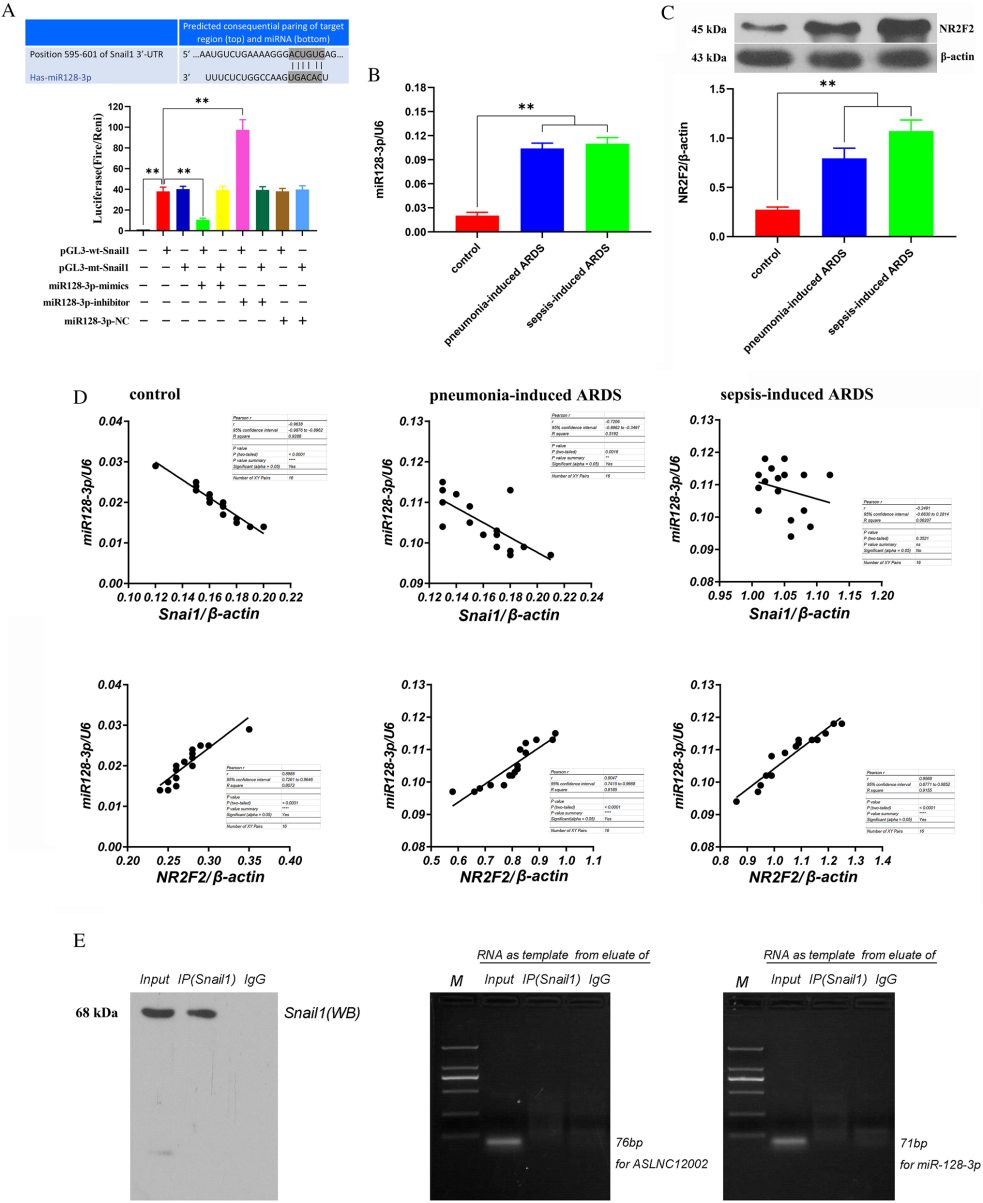
Correlation analysis between miR128-3p and Snail1 or NR2F2 protein and verification of their interaction. **A** Prediction of the binding sites of miR128-3p in the 3’-UTR of Snail1 mRNA and the intracellular luciferase activity was detected 48 h after co-transfection in 293 T cells. The relative activity of luciferase is expressed as the ratio of firefly luciferase to Renilla luciferase. **B** miR128-3p levels were assessed in AECIIs via RT-qPCR. **C** Western blotting for the protein detection of NR2F2 in AECIIs by western blotting. WB experiment in Fig. 1C and **C** comes from the same detection experiment of the same samples, so the two results had the common internal parameters β-actin. **D** Correlation analysis between miR128-3p and Snail1 or NR2F2 protein expression in AECIIs of three groups (*n* = 16). **E** RIP assay. Immunoprecipitation of Snail1 (left), agarose gel electrophoresis for PCR amplification of the predicted Snail1 binding sequence in ASLNC12002 (middle) and miR128-3p (right). All data are expressed as the mean ± s.d. from at least three replicate experiments. **P < 0.01

Bioinformatics analysis suggested that there is a 7-base-binding site 5’- ACUGUGA-3’ of miR128-3p in the 3’-UTR of Snail1 mRNA. Luciferase assay showed that both pGL3-wt-Snail1 and pGL3-mt-Snail1 could significantly upregulate the luciferase activity in 293 T cells 48 h after transfection (*P* < 0.01, vs. 293 T cells). MiR-137 mimics and inhibitor could suppress or enhance the luciferase activity in pGL3-wt-Snail1-transfected 293 T cells (*P* < 0.01, vs. pGL3-wt-Snail1-transfected 293 T cells), but had no significant effect on the luciferase in pGL3-mt-Snail1-transfected 293 T cells (*P* > 0.05, vs. pGL3-mt-Snail1-transfected 293 T cells). There was no effect of miR128-3p NC on luciferase in pGL3-wt-Snail1 or pGL3-mt-Snail1-transfected 293 T cells (*P* > 0.05, vs. pGL3-wt-Twist1 or pGL3-mt-Snail1-transfected 293 T cells) (Fig. 1A). RT-qPCR results showed that miR128-3p levels in AECIIs of patients with sepsis-induced ARDS and pneumonia-induced ARDS were significantly higher than that of control group (*P* < 0.01), but there was no difference of miR128-3p levels between pneumonia-induced ARDS group and sepsis-induced ARDS group (*P* > 0.05) (Fig. 2B). Moreover, western blotting results demonstrated that the NR2F2 protein expressions in AECIIs of patients with pneumonia-induced ARDS and sepsis-induced ARDS were upregulated markedly than that of control group (*P* < 0.01), but there was no difference in NR2F2 protein expressions between pneumonia-induced ARDS group and sepsis-induced ARDS group (*P* > 0.05) (Fig. 2C). The correlation analysis showed that miR128-3p was negatively correlated with the expression of Snail1 protein in AECIIs of control group and pneumonia-induced ARDS group, but not in sepsis-induced ARDS group. While, miR128-3p was positively correlated with the expression of NR2F2 protein in AECIIs of all groups (Fig. 2D). RIP results showed that neither ASLNC12002 nor miR128-3p directly bound to Snail1 protein (Fig. 2E). These results suggested that the increase of miR128-3p level in AECIIs of patients with ARDS was benefited from the increasing of NR2F2 protein. In addition, compared with the pneumonia-induced ARDS group, the further enhancement of Snail1 protein expression in AECIIs of patients with sepsis-induced ARDS is most likely due to the inactivation of miR128-3p function.

### ASLNC12002 is highly expressed and deactivates the effect of miR128-3p on Snail1 in AECIIs of patients with sepsis-induced ARDS

**Fig. 3:**
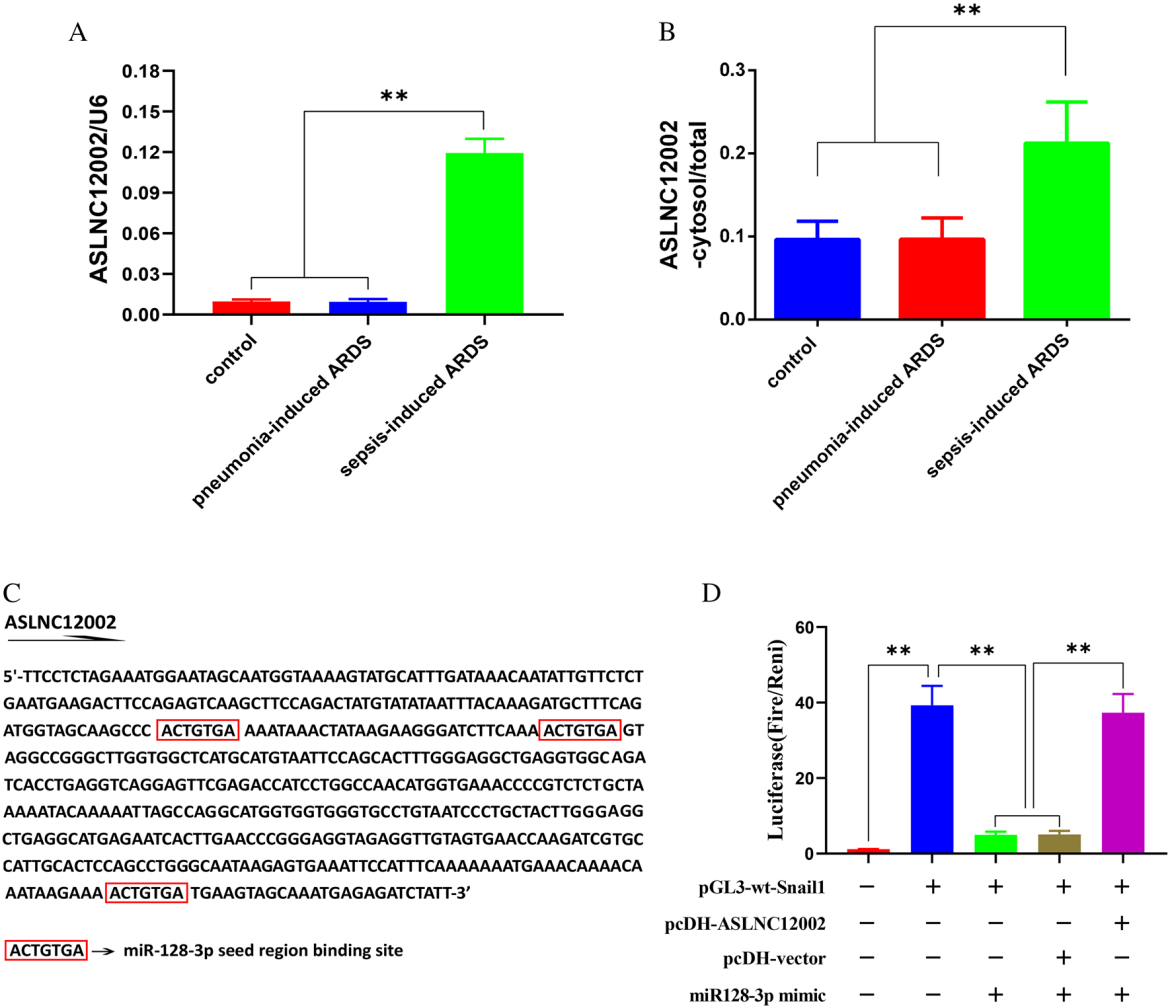
Validation of ASLNC12002 to competitively inhibit the binding of miR128-3p to the 3'UTR region of Snail1 mRNA. **A** Relative levels of ASLNC12002 in AECIIs were analyzed using RT-qPCR 72 h after lentivirus infection. U6 served as an internal reference, and the 2^−ΔCt^ method was used to analyze inter-group differences. **B** The proportion of ASLNC12002 in the cytoplasm to the total cells was analyzed by RT-qPCR. **C** Bioinformatics analysis of the binding sites of miR128-3p on ASLNC12002. **D** The luciferase activity was detected 48 h after co-transfection in 293 T cells. All data are expressed as the mean ± s.d. **P < 0.01

The results of RT- qPCR showed that the ASLNC12002 level in AECIIs of patients with sepsis-induced ARDS was significantly higher than that of control group and pneumonia-induced ARDS group (*P* < 0.01) (Fig. 3A). RT-qPCR also showed that ASLNC12002 accumulated more obviously in the cytoplasm of AECIIs in patients with sepsis-induced ARDS than in control group and pneumonia-induced ARDS group (*P* < 0.01) (Fig. 3B). Bioinformatics analysis showed that ASLNC12002 contained at least three binding sites (5’-ACTGTGA-3’) of miR128-3p (Fig. 3C). The luciferase assay showed that miR128-3p mimic significantly decreased the luciferase activity in 293 T cells transfected with pGL3-wt-Snail1, while the transfection of pcDH-ASLNC12002 reversed the effect of miR128-3p mimic on luciferase (Fig. 3D).

### Knockdown of ASLNC12002 inhibits the proliferation, invasion, and apoptosis of AECIIs in patients with sepsis-induced ARDS

**Fig. 4:**
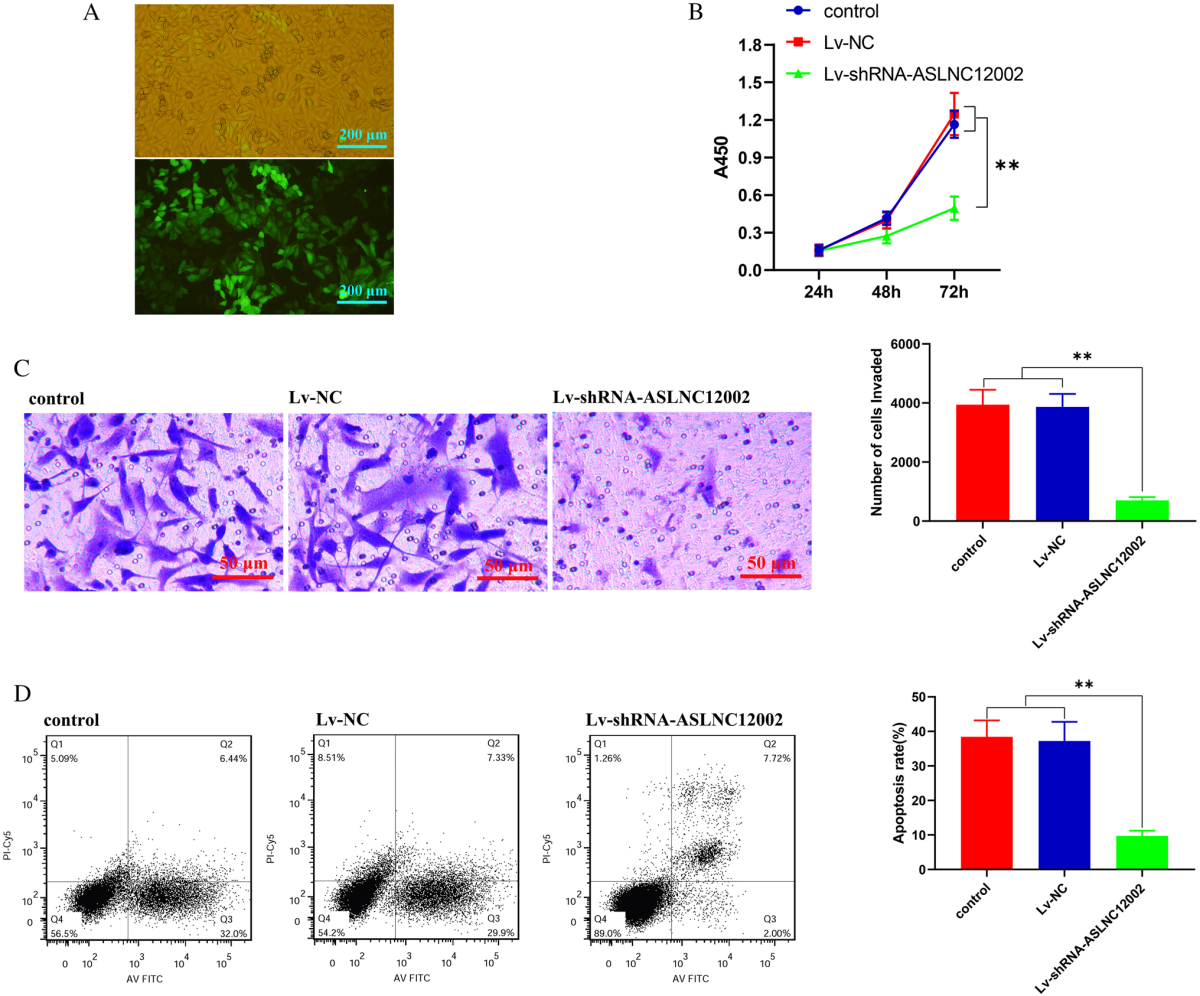
Analysis of cell proliferation, invasion, and apoptosis of AECIIs 72 h after lentivirus infection. **A** Infection efficiency analysis of lentiviruses in AECIIs. GFP expression in AECIIs 72 h following infection with Lv-shRNA-ASLNC12002 is presented (down). **B** Proliferation assay. The y-coordinate represents the absorbance at 450 nm of the test wells, and there is a strict linear relationship between the absorbance and proliferative activity of the cell samples. **C** Invasion assay. DAPI was used to stain the cells that passed through the membrane, and the cell counts were estimated based on the absorbance and a standard curve. The y-coordinate represents the number of invased AECIIs with different treatments. **D** The apoptosis rate was detected by flow cytometry. Left, the distribution map of cells in each stage of apoptosis; right, the differences of the apoptosis rate of cells between the three groups. The total apoptosis rate was calculated as the sum of the early and late apoptosis rates. All data are expressed as mean ± s.d. of at least three independent experiments. **P < 0.01

**Fig. 5:**
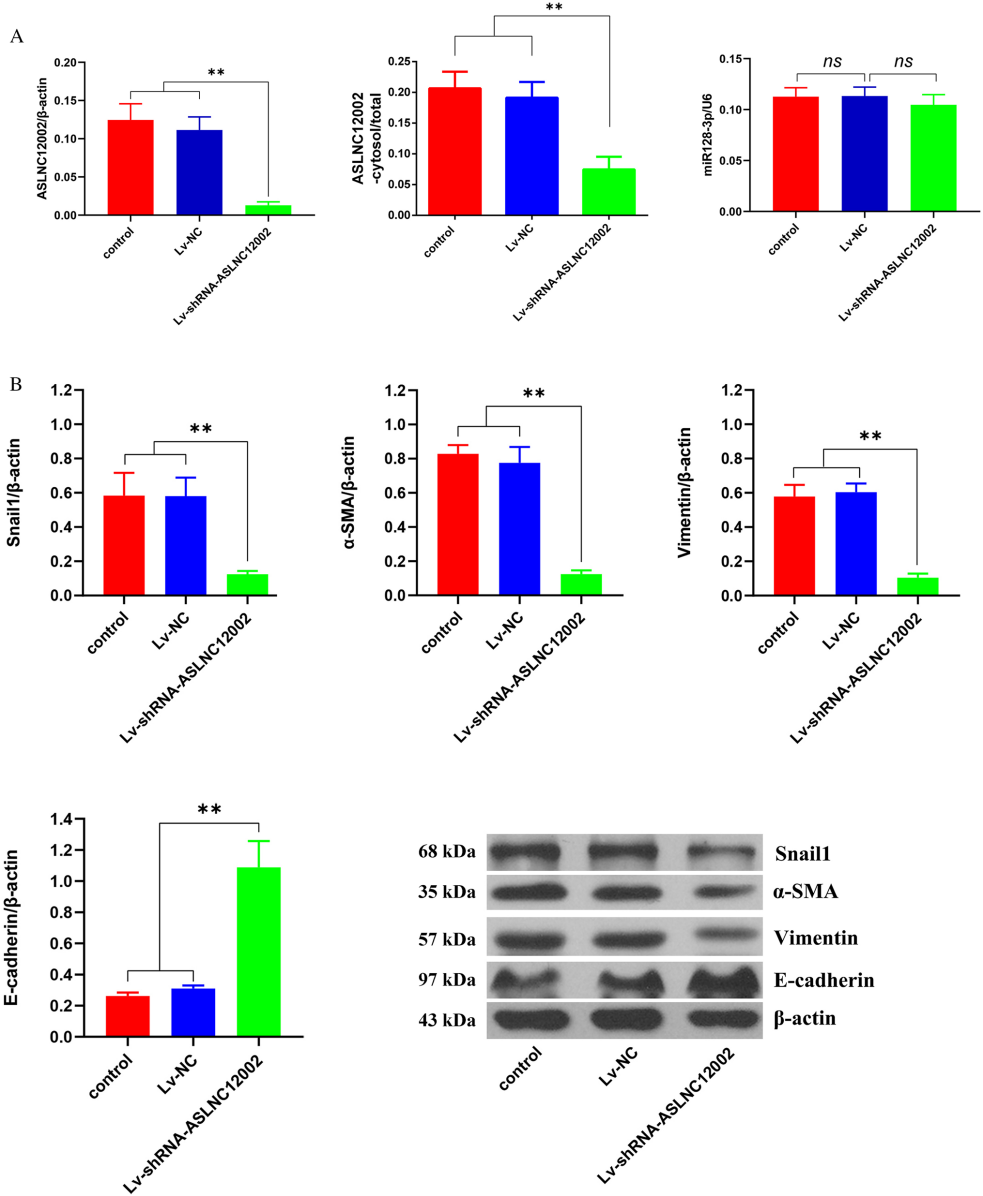
Evaluation of the effect of ASLNC12002 silencing on the EMT of AECIIs in patients with sepsis-induced ARDS by the detection of markers and functional proteins’ expression. **A** The relative contents of ASLNC12002 and miR128-3p, and the proportion of ASLNC12002 in cytoplasm were detected by RT-qPCR in AECIIs infected with recombinant virus (Lv-NC or Lv-shRNA-ASLNC12002) for 72 h. **B** The expressions of Snail1, α-SMA, Vimentin, and E-cadherin proteins were detected by western blotting in AECIIs infected with recombinant virus (Lv-NC or Lv-shRNA-ASLNC12002) for 72 h. Data are expressed as mean ± s.d. of at least three independent experiments. **P < 0.01

After being infected by lentiviruses for 72 h, the majority of cells in the same field of vision had GFP expression, indicating that the lentivirus infection efficiency on the cells was close to 100% (Fig. 4A). CCK-8 and invasion assays showed that compared with control group and Lv-NC group, the proliferation and invasion of AECIIs in Lv-shRNA-ASLNC12002 group were all decreased significantly (*P* < 0.01) (Fig. 4B, C). Meanwhile, Lv-shRNA-ASLNC12002 also caused a significant decrease of apoptosis (*P* < 0.01, vs. control or Lv-NC group) (Fig. 4D). The RT-qPCR showed that infection of Lv-shRNA-ASLNC12002 significantly decreased ASLNC12002 level and aggregation of in the nucleus (*P* < 0.01, vs. control or Lv-NC group), while had no effect on miR128-3p expression in AECIIs from patients with sepsis-induced ARDS (*P* > 0.05). In addition, RT-qPCR results also showed that ASLNC12002 knockdown could significantly reduce the proportion of ASLNC12002 content in AECIIs cytoplasm to the total in the cells (*P* < 0.01, vs. control or Lv-NC group) (Fig. 5A). Western blotting showed that the expressions of Snail1, α-SMA, and Vimentin proteins were decreased, but E-cadherin was increased significantly in AECIIs transfected with Lv-shRNA-ASLNC12002 (*P* < 0.01, vs. control or Lv-NC group) (Fig. 5B). The results prove that ASLNC12002 knockdown inhibits EMT of AECIIs in patients with sepsis-induced ARDS.

### ASLNC12002 regulates EMT in AECIIs by NR2F2/miR128-3p/Snail1 pathway

**Fig. 6:**
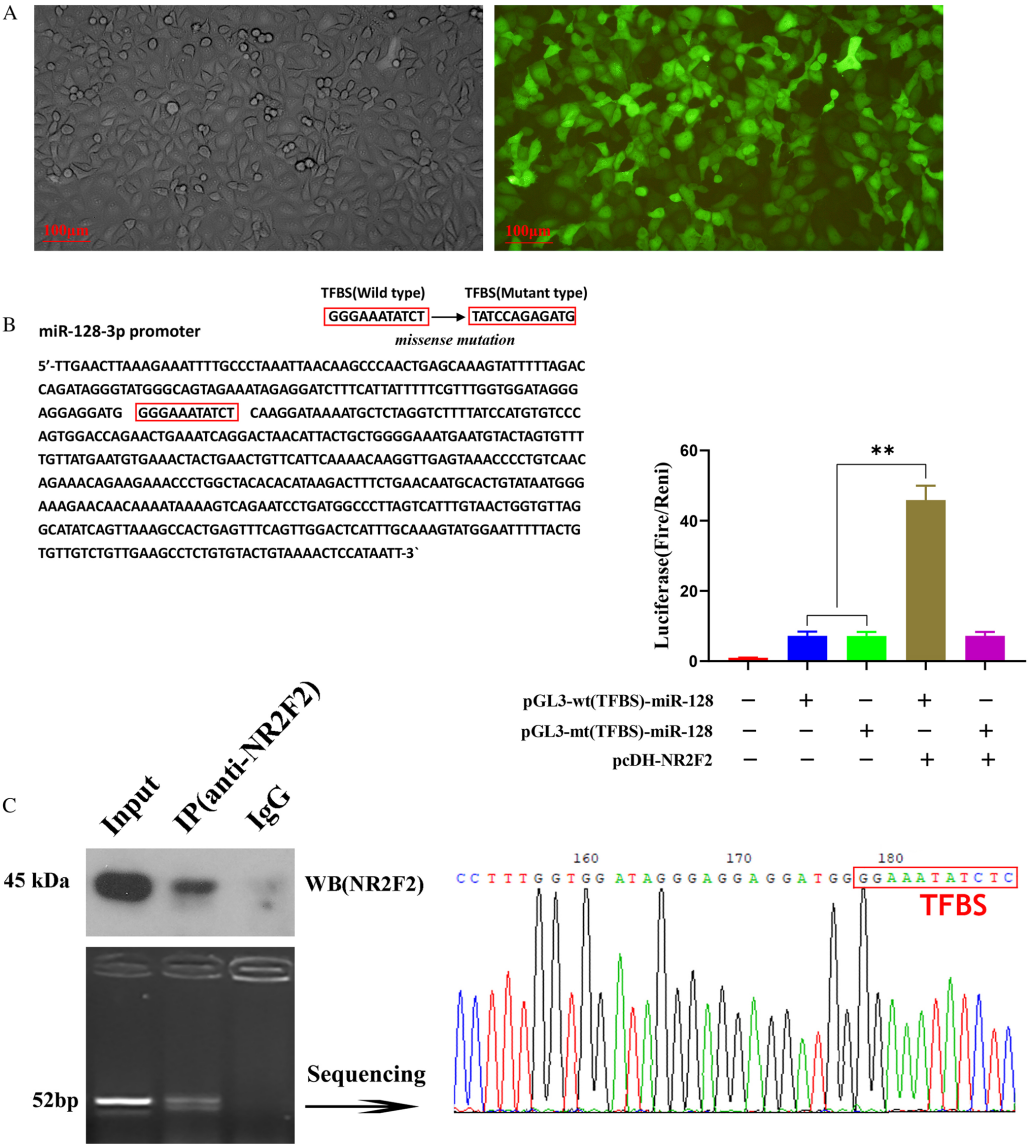
Verification of the effect of NR2F2 protein on miR128-3p transcription by binding to its promoter. **A** Verification of Predicted miR128-3p promoter activity. The GFP expressions of 293 T cells transfected with plasmids vector carrying miR128-3p promoter were detected by fluorescence microscope. **B** Prediction of the TFBS of NR2F2 in the miR128-3p promoter which were confirmed by luciferase assay. **C** The combination of miR-128 and NR2F2 was detected by Chip-PCR assay. The tests were carried out on three biological triplicates, and data are expressed as mean ± s.d. ** *P* < 0.01, * *P* < 0.05

Experimental results showed that there was obvious GFP expression in 293 T cells which were transfected with pcDH-pro(miR128) (Fig. 6A). Bioinformatics analysis showed that there was a TFBS of NR2F2 on miR128-3p promoter. The luciferase assay showed that transfection of pcDH-NR2F2 markedly increased the luciferase activity in 293 T cells transfected with pGL3-TFBS (wt)-miR128 (*P* < 0.01) (Fig. 6B). Chip-PCR assay showed that NR2F2 protein could bind to miR128-3p promoter through TBST (Fig. 6C). These results reveal that NR2F2 can regulate the transcription of miR128-3p by binding to miR128-3p promoter. As an independent factor, ASLNC12002 can promote EMT in AECIIs by inhibiting NR2F2/miR128-3p/Snail1 pathway via acting as the miR128-3p sponge.

## Discussion

**Fig. 7:**
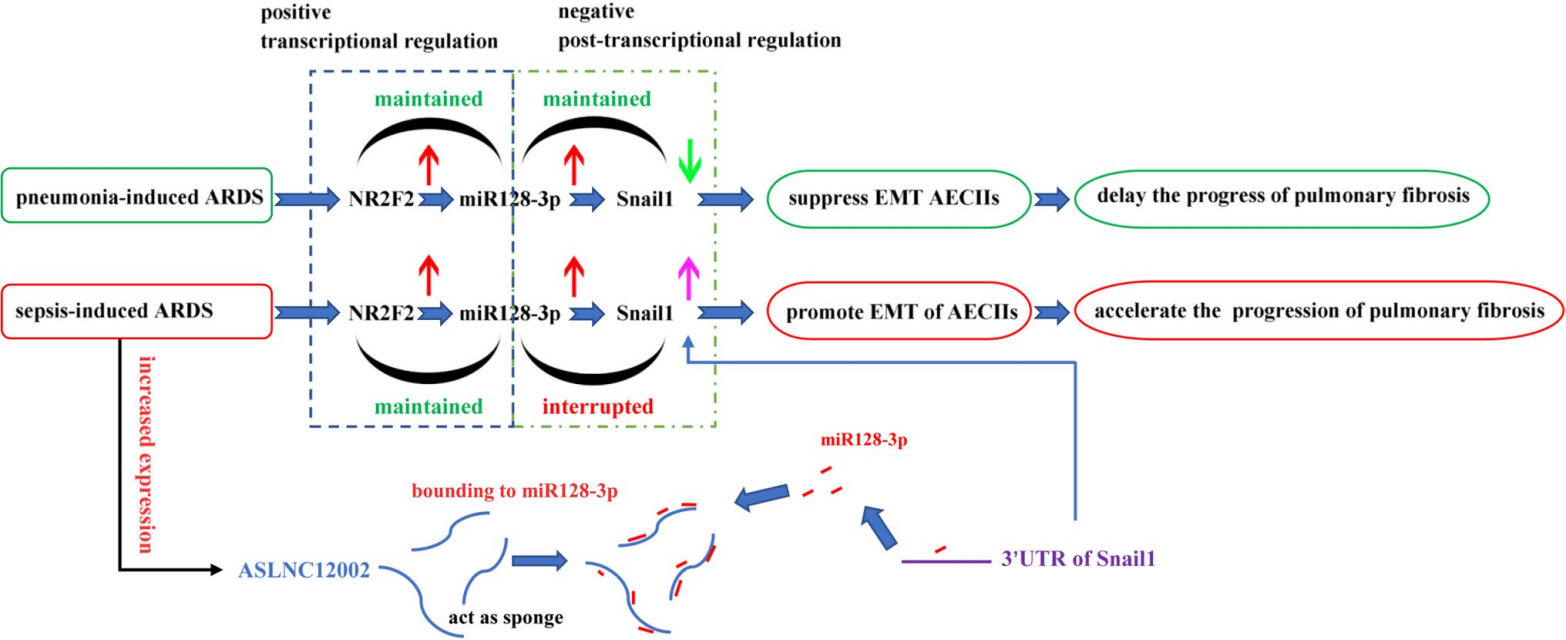
The graphical abstract. The mechanism hypothesis diagram for sepsis-induced ARDS

In this study, to our knowledge, we for the first time showed that ASLNC12002 knockdown inhibited EMT of AECIIs in patients with sepsis-induced ARDS by reactivating the NR2F2/miR128-3p/Snail1 pathway. Mechanically, we identified that ASLNC12002 promotes EMT of AECIIs in patients with sepsis-induced ARDS by competitively binding to miR128-3p with Snail1 3’UTR and suppressing NR2F2/miR128-3p/Snail1 signal pathway. Our study evaluated that, as an independent factor, the increased expression of ASLNC12002 may be the internal reason why patients with sepsis-induced ARDS are more likely to develop pulmonary fibrosis or early fibrosis compared to pneumonia-induced ARDS. The major findings of the study are as follows: (1) AECIIs of patients with sepsis-induced ARDS show an obvious EMT phenotype. (2) Up-regulation of Snail1 protein is caused by inactivation of miR128-3p in AECIIs of patients with sepsis-induced ARDS. (3) The highly expressed ASLNC12002 in AECIIs of patients with sepsis-induced ARDS inhibited the negative regulation of miR128-3p on Snail1 protein by acting as a sponge. (4) ASLNC12002 knockdown inhibits EMT of AECIIs in patients with sepsis-induced ARDS. Therefore, our results indicate that ASLNC12002 depletion may be a novel strategy for the prevention and treatment of pulmonary fibrosis of sepsis-induced ARDS (Fig. 7).

The incidence of pulmonary fibrosis is higher in patients with sepsis-induced ARDS than in patients with pneumonia-induced ARDS, and it is the important factor for poor prognosis of patients with sepsis-induced ARDS [20]. However, up to now, the reason why pulmonary fibrosis has different disease progression in different types of ARDS patients is not very clear. Several studies have shown that EMT of alveolar epithelial cells might be fundamental mechanism of pulmonary fibrosis. Pulmonary fibrosis could be urged to onset by inducing or promoting EMT of alveolar epithelial cells, and be expected to be cured by blocking the process of EMT and its signaling pathways [21, 22]. In this study, we collected the BALF from 48 patients used for AECIIs were sorting by flow cytometry. Compared to control group and pneumonia-induced ARDS group, the epithelial marker E-cadherin protein expression decreased, while the expressions of Snail1, Vimentin, and α-SMA proteins increased significantly in AECIIs of patients with sepsis-induced ARDS. Interestingly, mRNA expression changes of α-SMA, Vimentin, and E-cadherin were consistent with their proteins in AECIIs of all groups, while there was no significant difference in the mRNA levels of Snail among control group, pneumonia-induced ARDS group, and sepsis-induced ARDS group. These results indicate that the abnormal expression of Snail1 in AECIIs of patients with sepsis-induced ARDS is caused by inactivation of its post-transcriptional regulation mechanism.

As an important transcription factor, Snail1 plays a key positive regulatory effect in promoting EMT by downregulating E-cadherin expression and activating Vimentin and α-SMA in epithelial cells indirectly [23, 24]. By bioinformatics analysis and luciferase assay, we found that miR128-3p inhibited Snail expression by targeting 3’-UTR of Snail mRNA. This study also showed that miR128-3p levels in AECIIs of patients with ARDS, whether they were derived by pneumonia or sepsis, were significantly higher than that of control group, but there was no difference between pneumonia-induced ARDS group and sepsis-induced ARDS group. These results suggest that miR128-3p negatively regulated Snail protein by targeting 3’-UTR of Snail mRNA and the effect of miR128-3p on Snail1 protein is maintained only in AECIIs of control group and pneumonia-induced ARDS group. In other words, miR128-3p lost its inhibition on Snail1 protein expression in AECIIs of patients with sepsis-induced ARDS.

Recent studies have reported that lncRNA had an influence on the occurrence and development of pulmonary fibrosis by regulating miRNAs [25, 26]. In the current study, we found that there was no significant difference in the expression of lncRNA-ASLNC12002 between control group and pneumonia-induced ARDS group, while ASLNC12002 level in AECIIs of patients with sepsis-induced ARDS was significantly higher than those of control group and pneumonia-induced ARDS group. Further research showed that the deletion of ASLNC12002 in AECIIs of patients with sepsis-induced ARDS inhibited the proliferation, invasion, and apoptosis significantly. Besides, ASLNC12002 knockdown downregulated the expressions of proteins Snail1, α-SMA, and Vimentin, and upregulated E-cadherin protein expression significantly. These results provide the evidence that ASLNC12002 level upregulated significantly in AECIIs of patients with sepsis-induced ARDS and ASLNC12002 knockdown inhibits EMT and the proliferation, invasion, and apoptosis of AECIIs. Hence, it is essential to reveal new detailed mechanisms and molecular pathways about ASLNC12002 in sepsis-induced ARDS and pulmonary fibrosis.

Notably, accumulating evidence has suggested that lncRNA can act as competing endogenous RNAs or “RNA sponges”, interacting with microRNAs in a manner that can sequester these molecules and reduce their regulatory effect on target mRNAs [27, 28]. Studies have shown that lncRNA H19 promotes bleomycin-(BLM) induced pulmonary fibrosis by regulating the miR-196a/COL1A1 pathway [29]. Besides, lncRNA CYTOR sponges miR-195 to modulate proliferation, migration, invasion, and radio-sensitivity in non-small cell lung cancer cells [30]. Consistently, our results of RIP confirmed that ASLNC12002 was not directly combined with Snail1 (data not shown). Bioinformatics analysis and luciferase reporter assay showed that ASLNC12002 sequence contained at least three binding sites of miR128-3p and overexpression of ASLNC12002 blocked the effect that miR128-3p inhibited Snail1 by targeting its 3’-UTR. These results reveal that ASLNC12002 can combine with miR128-3p and deactivate the effect of miR128-3p in AECIIs of patients with sepsis-induced ARDS.

NR2F2, the nuclear receptor subfamily 2, group F, member 2, regulates a whole of important signal pathways [31, 32], and NR2F2 is one of the main regulatory factors in EMT and closely related to invasion and migration of tumor and poor prognosis of patients [33]. Although the role of NR2F2 has been explored in a few studies, our study is for the first time to analyze its role and mechanism in AECIIs of patients with sepsis-induced ARDS. Here, we first found that the NR2F2 protein expression was upregulated markedly in AECIIs whether they were derived from patients with pneumonia-induced ARDS or sepsis-induced ARDS. More importantly, it was confirmed that miR128-3p promoter contained the TFBS of NR2F2 using bioinformatics analysis. Furthermore, luciferase and Chip-sequencing verified that NR2F2 could positively regulate the transcription of miR128-3p by binding to the TFBS in its promoter. These results suggest thatNR2F2/miR128-3p/Snail1 pathway exists in EMT progression of AECIIs in ARDS patients with sepsis-induced ARDS, and it has the basic attribute of inhibition on EMT. It remains further exploration about the mechanism of ASLNC12002 regulating EMT by NR2F2/miR128-3p/Snail1 axis. As an independent factor, ASLNC12002 inhibits EMT progression by inactivating NR2F2/miR128-3p/Snail1 axis depending upon its functional inactivation of miR128-3p by acting as a sponge. However, it remains further exploration about the mechanism of the increased expression of ASLNC12002 in AECIIs of patients with sepsis-induced ARDS. In conclusion, our findings describe a novel mechanism for pulmonary fibrosis that involves EMT of AECIIs in patients with sepsis-induced ARDS. The present study demonstrates that ASLNC12002 regulates EMT by NR2F2/miR128-3p/Snail1 pathway and knockdown of ASLNC12002 inhibits EMT, thus indicating that ASLNC12002 may be a potential target for therapy of sepsis-induced ARDS and its related products (such as degradation residues, encoded polypeptides, etc.) have the potential to become diagnostic and prognostic biomarkers for patients with sepsis-induced ARDS.

## Supplementary Information

Below is the link to the electronic supplementary material.

Supplementary file1 (PDF 50 KB)

## Data Availability

The datasets used and/or analyzed during the current study are available from the corresponding author on reasonable request.

## Abbreviations

ARDS: Acute respiratory distress syndrome
LPS: Lipopolysaccharide
EMT: Epithelial-mesenchymal transition
AECIIs: Type II alveolar epithelial cells
NR2F2: Nuclear receptor subfamily 2 group F member 2
lncRNAs: Long non-coding RNAs
miR: Micro RNA
BALF: Bronchoalveolar lavage fluids
UTR: Untranslated regions
TFBS: Transcription factor-binding site

## References

[1] Mulchandani N, Yang WL, Khan MM (2015). Stimulation of brain AMP-activated protein kinase attenuates inflammation and acute lung injury in sepsis. Mol Med (Cambridge, Mass).

[2] Seymour CW, Liu VX, Iwashyna TJ (2016). Assessment of clinical criteria for sepsis: for the third international consensus definitions for sepsis and septic shock (Sepsis-3). JAMA.

[3] van der Poll T, van de Veerdonk FL, Scicluna BP, Netea MG (2017). The immunopathology of sepsis and potential therapeutic targets. Nat Rev Immunol.

[4] Mira JC, Gentile LF, Mathias BJ (2017). Sepsis pathophysiology, chronic critical illness, and persistent inflammation-immunosuppression and catabolism syndrome. Crit Care Med.

[5] Burnham EL, Janssen WJ, Riches DW, Moss M, Downey GP (2014). The fibroproliferative response in acute respiratory distress syndrome: mechanisms and clinical significance. Eur Respir J.

[6] Suzuki T, Tada Y, Gladson S (2017). Vildagliptin ameliorates pulmonary fibrosis in lipopolysaccharide-induced lung injury by inhibiting endothelial-to-mesenchymal transition. Respir Res.

[7] Tian R, Zhu Y, Yao J (2017). NLRP3 participates in the regulation of EMT in bleomycin-induced pulmonary fibrosis. Exp Cell Res.

[8] Cao Y, Liu Y, Shang J (2019). Ang-(1–7) treatment attenuates lipopolysaccharide-induced early pulmonary fibrosis. Lab Invest.

[9] Cao Y, Liu Y, Ping F, Yi L, Zeng Z, Li Y (2018). miR-200b/c attenuates lipopolysaccharide-induced early pulmonary fibrosis by targeting ZEB1/2 via p38 MAPK and TGF-beta/smad3 signaling pathways. Lab Invest.

[10] Baulida J, Díaz VM, Herreros AG (2019). Snail1: a transcriptional factor controlled at multiple levels. J Clin Med.

[11] Gholami MD, Falak R, Heidari S (2019). A truncated snail1 transcription factor alters the expression of essential EMT markers and suppresses tumor cell migration in a human lung cancer cell line. Recent Pat Anticancer Drug Discov.

[12] Sanchez Calle A, Kawamura Y, Yamamoto Y, Takeshita F, Ochiya T (2018). Emerging roles of long non-coding RNA in cancer. Cancer Sci.

[13] Kazemzadeh M, Safaralizadeh R, Orang AV (2015). LncRNAs: emerging players in gene regulation and disease pathogenesis. J Genet.

[14] Guzel E, Okyay TM, Yalcinkaya B, Karacaoglu S, Gocmen M, Akcakuyu MH (2020). Tumor suppressor and oncogenic role of long non-coding RNAs in cancer. North Clin Istanb.

[15] Wang W, Yang N, Wen R, Liu CF, Zhang TN (2021). Long noncoding RNA: regulatory mechanisms and therapeutic potential in sepsis. Front Cell Infect Microbiol.

[16] Wang HR, Guo XY, Liu XY, Song X (2020). Down-regulation of lncRNA CASC9 aggravates sepsis-induced acute lung injury by regulating miR-195–5p/PDK4 axis. Inflamm Res.

[17] Qiu N, Xu X, He Y (2020). LncRNA TUG1 alleviates sepsis-induced acute lung injury by targeting miR-34b-5p/GAB1. BMC Pulm Med.

[18] Cui C, Chen X, Du W (2020). Correlations of inflammation, oxidative stress and prognosis with expression of LncRNA H19 in rats with sepsis-evoked lung injury. Panminerva Med.

[19] Huang X, Zhao M (2019). High expression of long non-coding RNA MALAT1 correlates with raised acute respiratory distress syndrome risk, disease severity, and increased mortality in sepstic patients. Int J Clin Exp Pathol.

[20] Zhou WQ, Wang P, Shao QP, Wang J (2016). Lipopolysaccharide promotes pulmonary fibrosis in acute respiratory distress syndrome (ARDS) via lincRNA-p21 induced inhibition of Thy-1 expression. Mol Cell Biochem.

[21] Qu H, Liu L, Liu Z (2019). Blocking TBK1 alleviated radiation-induced pulmonary fibrosis and epithelial–mesenchymal transition through Akt-Erk inactivation. Exp Mol Med.

[22] Felton VM, Borok Z, Willis BC (2009). N-acetylcysteine inhibits alveolar epithelial–mesenchymal transition. Am J Physiol Lung Cell Mol Physiol.

[23] Wu D, Zhao B, Qi X (2018). Nogo-B receptor promotes epithelial–mesenchymal transition in non-small cell lung cancer cells through the Ras/ERK/Snail1 pathway. Cancer Lett.

[24] You J, Li M, Cao LM (2019). Snail1-dependent cancer-associated fibroblasts induce epithelial–mesenchymal transition in lung cancer cells via exosomes. QJM.

[25] Jiang H, Chen Y, Yu T (2018). Inhibition of lncRNA PFRL prevents pulmonary fibrosis by disrupting the miR-26a/smad2 loop. Am J Physiol Lung Cell Mol Physiol.

[26] Liu X, Gao S, Xu H (2018). lncRNAPCAT29 inhibits pulmonary fibrosis via the TGFbeta1regulated RASAL1/ERK1/2 signal pathway. Mol Med Rep.

[27] Aftab MN, Dinger ME, Perera RJ (2014). The role of microRNAs and long non-coding RNAs in the pathology, diagnosis, and management of melanoma. Arch Biochem Biophys.

[28] Paraskevopoulou MD, Hatzigeorgiou AG (2016). Analyzing MiRNA-LncRNA Interactions. Methods Mol Biol (Clifton, NJ).

[29] Lu Q, Guo Z, Xie W (2018). The lncRNA H19 mediates pulmonary fibrosis by regulating the miR-196a/COL1A1 axis. Inflammation.

[30] Zhang J, Li W (2018). Biosci Rep.

[31] Zhang C, Han Y, Huang H, Qu L, Shou C (2014). High NR2F2 transcript level is associated with increased survival and its expression inhibits TGF-β-dependent epithelial–mesenchymal transition in breast cancer. Breast Cancer Res Treat.

[32] Polvani S, Pepe S, Milani S, Galli A (2019). COUP-TFII in health and disease. Cells.

[33] Zhao G, Weiner AI, Neupauer KM (2020). Regeneration of the pulmonary vascular endothelium after viral pneumonia requires COUP-TF2. Sci Adv.

