# Supplementary figures and images for: Knockdown of lncRNA-ASLNC12002 alleviates epithelial–mesenchymal transition of type II alveolar epithelial cells in sepsis-induced acute respiratory distress syndrome
Source: Hum Cell. 2022 Dec 7;36(2):568–82. doi: 10.1007/s13577-022-00837-8 (PMC9734367; doi:10.1007/s13577-022-00837-8)

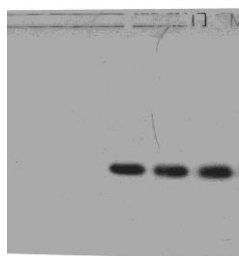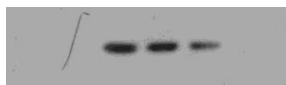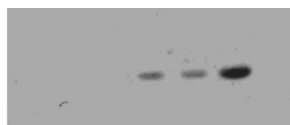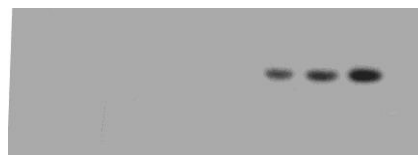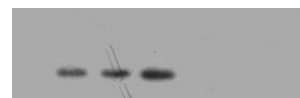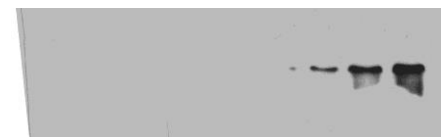

Fig1C, 2C

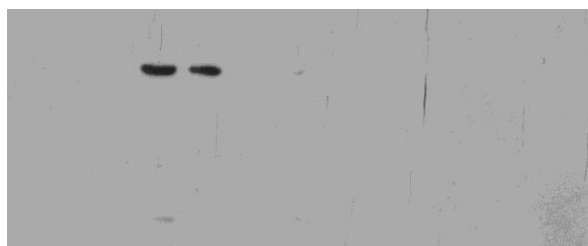

Fig2E

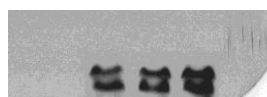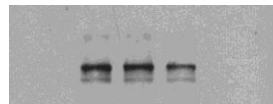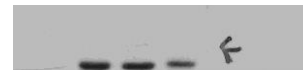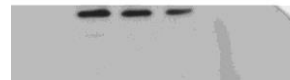

Fig5B

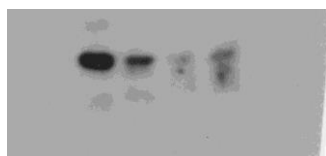

Fig6C

Supplement: Supplementary file 1 — Supplementary file1 (PDF 50 KB) [file 13577_2022_837_MOESM1_ESM.pdf]
